# A Specialized Dehydrogenase Provides l‐Phenyllactate for FR900359 Biosynthesis

**DOI:** 10.1002/cbic.202100569

**Published:** 2021-12-09

**Authors:** Sophie Klöppel, René Richarz, Daniel A. Wirtz, Natalia Vasenda, Gabriele M. König, Max Crüsemann

**Affiliations:** ^1^ Institute of Pharmaceutical Biology University of Bonn Nussallee 6 53115 Bonn Germany

**Keywords:** biosynthesis, *Chromobacterium vaccinii*, dehydrogenase, natural products, phenyllactic acid

## Abstract

d‐Phenyllactate (PLA) is a component of the selective Gq protein inhibitor and nonribosomal cyclic depsipeptide FR900359 (FR). Here we report a detailed biochemical investigation of pla biosynthesis and its incorporation into the natural product FR. The enzyme FrsC, member of the lactate/malate dehydrogenase superfamily, was shown to catalyze the formation of l‐PLA from phenylpyruvate. FrsC was kinetically characterized and its substrate specificity determined. Incorporation of l‐PLA was probed by assaying the adenylation domain FrsE‐A3 and feeding studies with a *Chromobacterium vaccinii* Δ*frsC* mutant, confirming preferred activation of l‐PLA followed by on‐line epimerization to d‐pla. Finally, detailed bioinformatic analyses of FrsC revealed its close relation to malate dehydrogenases from primary metabolism and suggest extensions in the substrate binding loop to be responsible for its adaptation to accepting larger aromatic substrates with high specificity.

## Graphical abstract

**The dehydrogenase FrsC** is involved in biosynthesis of the Gq protein inhibiting natural product FR900359. Here, we biochemically characterized FrsC, catalyzing the formation of l‐phenyllactate, and its subsequent incorporation into the FR assembly line followed by epimerization. A detailed bioinformatic analysis revealed that FrsC has evolved from primary metabolic enzymes to a specialized reductase accepting only aromatic substrates.

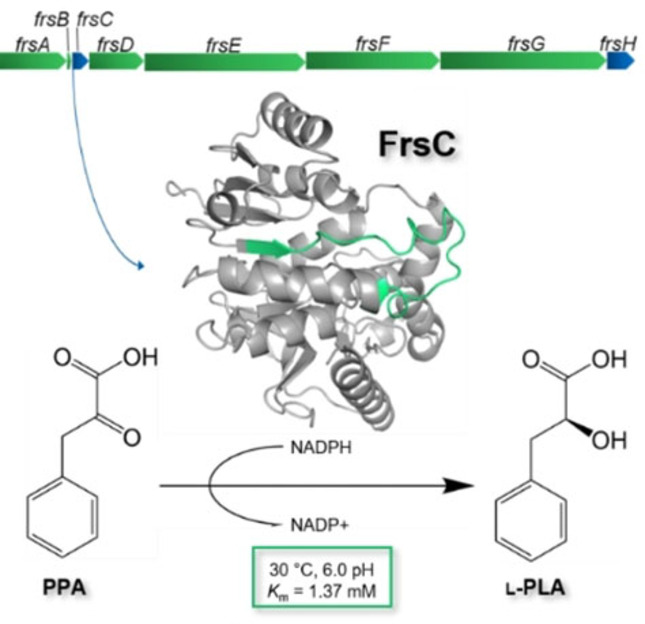

## Introduction

2‐Hydroxy‐3‐phenylpropanoic acid, also termed phenyllactic acid (PLA), is a natural organic acid known to be produced by lactic acid bacteria (LAB).[^1^, ^2^, ^3^] PLA owns a chiral carbon atom, forming two possible enantiomers, d‐pla and l‐PLA.^[4]^ In recent years, numerous LAB[^1^, ^5^, ^6^, ^7^, ^8^, ^9^] and non‐LAB[^4^, ^9^] with the ability to produce PLA were characterized. PLA and its derivative 4‐hydroxyphenyllactic acid derive from phenylalanine metabolism. In order to avoid intracellular accumulation, phenylalanine is converted by a transaminase to phenylpyruvic acid (PPA), which may be further reduced to both PLA enantiomers by members of the l‐lactate and d‐lactate dehydrogenase families (l‐LDH, EC 1.1.1.27, d‐LDH, EC 1.1.1.28), respectively.[^2^, ^10^]

**Figure 1:**
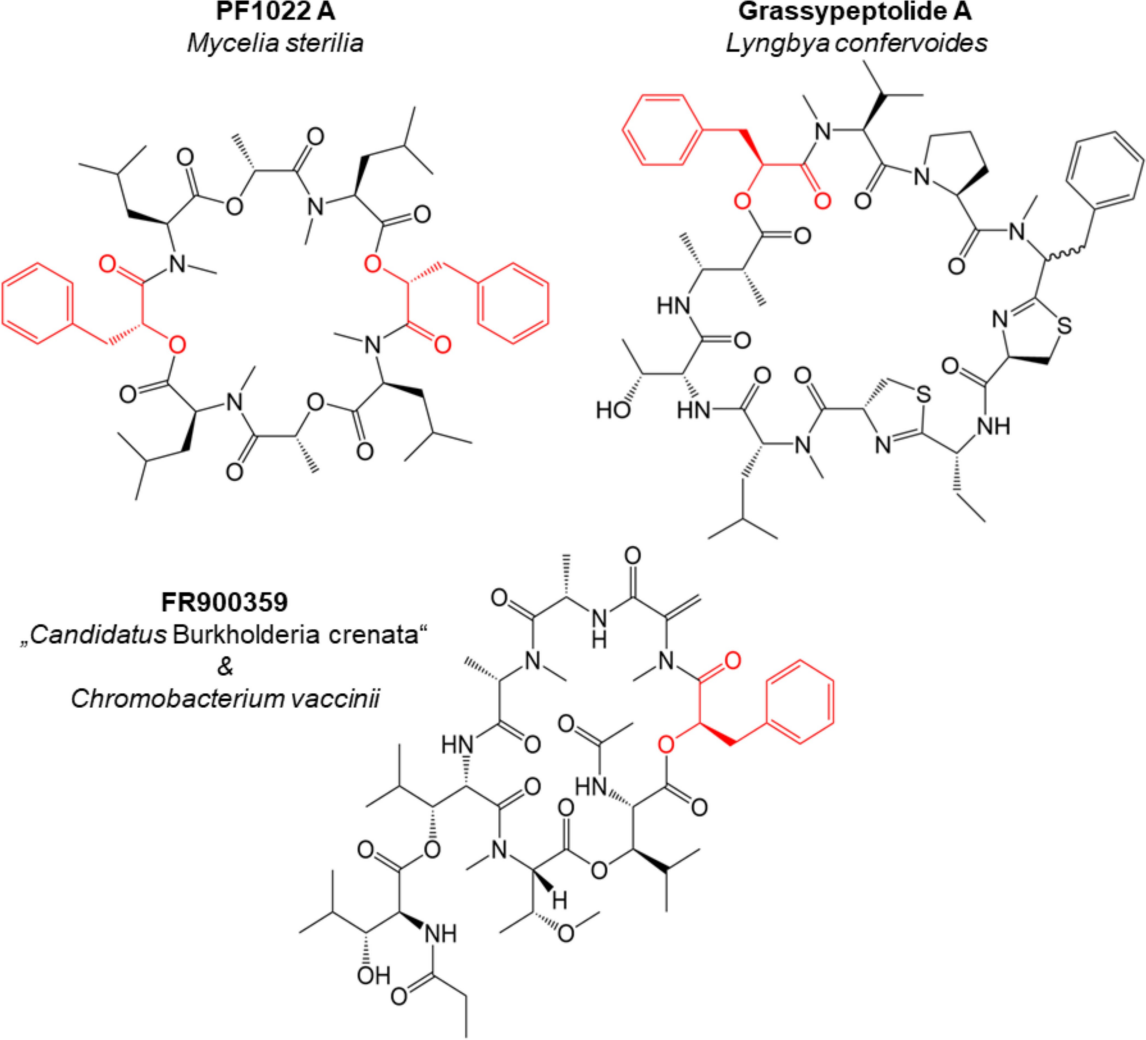
Structures of selected cyclic depsipeptide natural products containing phenyllactic acid (PLA), marked in red.

PLA is also a component of 21 known cyclic depsipeptide (CD) natural products (SciFinder search 6/2021). CDs are defined as peptide macrocycles, which contain at least one ester linkage^[11]^ and are produced by a broad range of organisms such as (cyano)bacteria, fungi, algae, and plants, displaying a wide spectrum of biological activities (antitumor, antibacterial, antifungal, insecticidal, and anthelmintic).[^11^, ^12^] Bacteria and fungi are known to synthesize CDs through non‐ribosomal peptide synthetases (NRPS), giant multi‐domain assembly‐line enzyme complexes.^[11]^ Three examples of CDs containing PLA moieties are shown in Figure 1. The *N*‐methylated PF1022A‐D, synthesized by an undefined fungus, i. e. *Mycelia sterilia*, contain up to four PLA residues[^13^, ^14^] and possess strong activity (oral treatment 1–10 mg/kg^[15]^) against nematodes and a low toxicity to the host animals,[^16^, ^17^] making them effective anthelmintic drugs.^[18]^ Grassypeptolides A–C, originating from the marine cyanobacterium *Lyngbya confervoides* are also most likely assembled by an NRPS system, although no biosynthetic gene cluster (BGC) has yet been described. Grassypeptolides show high cytotoxicity against a variety of human cancer cell lines due to inhibitory effects on dipeptidyl peptidase 8 (DPP8) and T‐cell activation.[^19^, ^20^]

The structurally complex CD FR900359 (FR), member of a small natural product family that we termed chromodepsins,^[21]^ was first isolated and characterized in 1988 from the higher plant *Ardisia crenata*.^[22]^ FR is a strong and selective inhibitor of heterotrimeric Gq proteins.^[23]^ These Gq proteins transmit signals from activated G protein‐coupled receptors (GPCRs) into the cell interior. Gq protein‐regulated signaling pathways are responsible for essential processes such as stimulus perception, inflammatory processes, targeted taxis, endo‐ and exocytosis, cell growth and cell differentiation in eukaryotic cells.^[24]^ Due to its Gq‐inhibiting function, FR is an excellent tool for studying the influence of G proteins on cellular signaling pathways and is also a promising candidate for the therapy of Gq‐protein‐related diseases. For example, FR effectively achieves bronchial relaxation in asthmatic lung diseases in a mouse model when applied locally.^[25]^ In addition, FR inactivates mutated heterotrimeric Gαq/11 proteins in uveal melanoma, enabling targeted and effective treatment of this cancer.[^26^, ^27^, ^28^] Furthermore, FR displays insect toxicity. Exposure of bean bug (*Riptortus pedestris*) nymphs to FR resulted in death and prevention of molting, indicating a beneficial role of FR for *A. crenata* against predators.^[29]^

**Figure 2:**
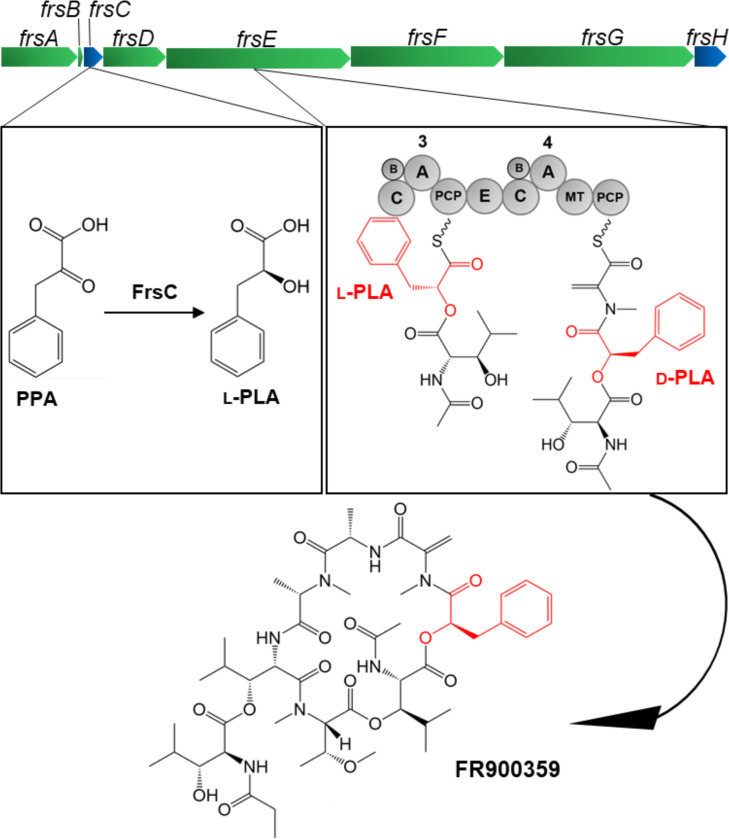
FR biosynthesis derived from bioinformatic analysis and *in vitro* experiments with focus on the formation and incorporation of phenyllactic acid (PLA). The genes encoding the NRPS FrsA‐FrsG are shown in green and the genes encoding modifying enzymes are depicted in blue. Highlighted is the reduction of phenylpyruvic acid (PPA) to l‐PLA, catalyzed by the dehydrogenase FrsC. l‐PLA is subsequently incorporated and epimerized by the FrsE module 3 (C=condensation domain, A=adenylation domain, PCP=peptidyl carrier protein, TE=thioesterase domain, E=epimerization domain, MT=methyltransferase domain, B=MbtH‐like protein FrsB). Finally, the natural product FR is shown, in which d‐PLA is marked in red.

Due to the structural peculiarities of FR,^[30]^ a bacterial origin was assumed. Indeed, further studies showed that it is produced by the uncultured bacterial endophyte “*Candidatus* Burkholderia crenata” and encoded by the *frs* BGC (Figure 2), which was proven responsible for FR biosynthesis.[^29^, ^31^] We recently characterized a second *frs* BGC in the cultivable soil bacterium *Chromobacterium vaccinii*, which produces FR under laboratory conditions, facilitating our studies on FR biosynthesis. The *frs* BGC from *C. vaccinii* is very similar to that in “*Ca*. B. crenata” regarding gene arrangement and domain architecture, showing overall sequence identity of approximately 70 %.^[32]^

The peptide backbone of FR is synthesized by the heptamodular NRPS FrsD‐G and subsequently decorated with the functionally important side chain by intermolecular transesterification mediated by the monomodular NRPS FrsA. The *frs* BGC also encodes an MbtH‐like protein (MLP), FrsB. MLPs are small, highly conserved proteins, named after MbtH from mycobactin biosynthesis.^[33]^ It has been shown that MLPs interact with adenylation (A) domains and are in many cases crucial for NRPS activity.^[34]^ In FR biosynthesis, we were able to prove the dependence of the FrsA and FrsD A domains on FrsB for catalytic activity.^[32]^

d‐pla is crucial for the potent bioactivity of chromodepsins, as shown by structure‐relationship studies on synthetic analogues of the structurally closely related Gq inhibitor YM‐254890 (YM). Modification of d‐pla to phenylalanine in YM‐3 resulted in 107‐fold loss of Gq‐inhibiting activity,^[35]^ while a change to d‐leucic acid in YM‐31 reduced Gq inhibition 25‐fold.^[36]^ According to our biosynthetic model, l‐PLA is incorporated into FR by the A domain of FrsE module 3 (Figure 2). We further postulated, that the epimerization domain present in FrsE3 would catalyze the subsequent conversion to d‐PLA.

Our preliminary bioinformatics analysis classified FrsC as a NAD(P)H‐dependent dehydrogenase with high sequence similarity to enzymes from primary metabolism such as malate dehydrogenases (MDH; EC 1.1.1.37) and lactate dehydrogenases.^[29]^ Both catalyze the NAD(P)+/NAD(P)H‐dependent oxidoreduction of 2‐keto and 2‐hydroxy carboxylic acids, leading to our hypothesis that FrsC is responsible for the reduction of PPA to l‐PLA.

Here, we biochemically characterize FrsC in detail, revealing the catalytic formation of l‐PLA. Further experimental data support our hypothesis of l‐PLA adenylation, thioesterification and subsequent epimerization by the third module of the FR assembly line. A detailed bioinformatic analysis of FrsC provides insight into its relation to dehydrogenases from primary metabolism and its evolution to accept the aromatic substrate phenylpyruvate with high specificity.

## Results and Discussion

**Figure 3:**
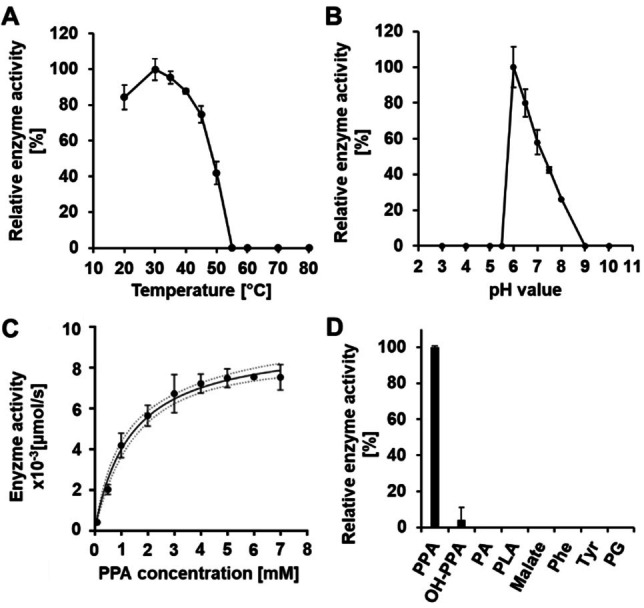
Biochemical characterization of FrsC. Determination of A) optimal temperature, B) optimal pH value, C) kinetic parameters with PPA as substrate (dashed lines=95 % confidence interval), D) substrate specificity, tested substrates were phenylpyruvic acid (PPA), 4‐OH‐phenylpyruvic acid (OH‐PPA), pyruvic acid (PA), phenyllactic acid (PLA), malate, l‐phenylalanine (Phe), l‐tyrosine (Tyr), and phenylglyoxylate (PG) (n=3).

To investigate its function as a putative PPA reductase, *frsC* from *C. vaccinii* was cloned into pET28 and overexpressed with an *N*‐terminal hexahistidinyl tag in *E. coli* BL21 (DE3) (Figure S1). Subsequently, the tagged enzyme was purified, and tested in an NAD(P)H‐dependent activity assay. Therefore, 1 μM of purified FrsC was incubated with 2 mM PPA and 0.2 mM NAD (P) H. The consumption of NAD (P) H was measured in a photometric assay at 340 nm. Compared to NADPH, using NADH as cofactor resulted in only 48 % relative activity. Thus, NADPH was used as coenzyme in further experiments. Subsequently, the optimal assay conditions were assessed by monitoring NADPH conversion at different temperatures (20–80 °C) and pH values (pH 3–10). FrsC showed the highest activity at 30 °C (Figure 3A). Above this optimum, the enzyme activity first slowly, and then rapidly decreased with increasing temperature. At temperatures of higher than 50 °C, no activity was detected. The maximum activity of FrsC was measured at a pH value of 6 (Figure 3B). With increasing pH, an almost linear decrease in activity was observed. FrsC did not show any activity in more acidic (< pH 5.5) or basic (> pH 9) environment. Thus, the optimal reaction conditions were defined as pH 6 and 30 °C. For evaluation of the kinetic parameters of this reaction, the hexahistidinyl tag was removed after protein purification by incubation with TEV protease to exclude any influence of the tag on the reaction (Figure S2). Next, the assay was performed with varying concentrations of PPA between 0.1 and 7 mM (1 μM FrsC, 0.5 mM NADPH). Applying the Michaelis‐Menten equation and a Hanes‐Woolf plot, a *K*_M_ of 1.37±0.41 mM, a V_max_ of 15.93±1.34 μmol min^−1^ mg^−1^, and a *k*_cat_ of 9.40±0.79 s^−1^ was determined, assuming that 100 % of the enzyme is active (Figure 3C, Figure S3, Table S1). We also tested 4‐OH‐phenylpyruvic acid (OH‐PPA), pyruvic acid (PA), PLA, malate, l‐phenylalanine (Phe), l‐tyrosine (Tyr) and phenylglyoxylate (PG) as alternative substrates under optimal reaction conditions (Figure 3D). Only 4‐OH‐PPA was converted with 4 % relative enzyme activity. These results indicate a high substrate specificity of FrsC with possible tolerances towards alteration only at the aromatic moiety. The identity of the reaction product was confirmed as PLA via high resolution HPLC‐MS analysis and comparison with a PLA standard (Figure S4).

**Figure 4:**
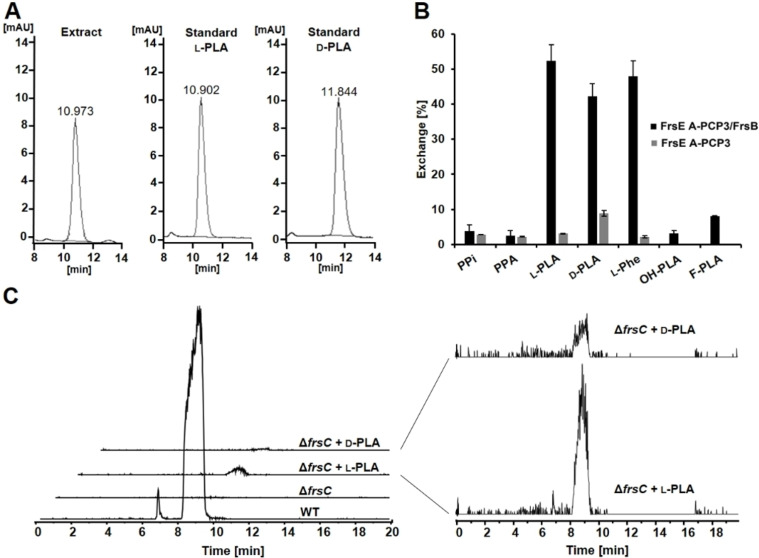
A) Identifying the configuration of the reaction product of FrsC: Chromatograms of the HPLC measurement of the organic *in vitro* assay extract, l‐PLA standard and d‐PLA standard with the corresponding retention times (230 nm). B) Results of the γ^18^O_4_ ATP exchange assay with FrsE A‐PCP3 in presence (black) and absence (grey) of FrsB evaluating the substrates phenylpyruvic acid (PPA), l‐phenyllactic acid (l‐PLA), d‐phenyllactic acid (d‐PLA), l‐phenylalanine (l‐Phe), dl‐p‐OH‐phenyllactic acid (OH‐PLA) and p‐fluoro‐d‐phenyllactic acid (F‐PLA), (n=3). C) Left: Extracted ion chromatograms (EIC) for *m/z*: 1002.54 of the *n‐*butanolic extracts of *C. vaccinii* wild type (WT), the *C. vaccinii* Δ*frsC*::FRT mutant and the feeding experiments of the mutant with l‐PLA and d‐PLA. Right: Direct comparison of EICs for *m/z*: 1002.54 of the extracts from feeding experiments with l‐PLA and d‐PLA.

Next, we aimed at determining the configuration of the FrsC reaction product PLA using HPLC with (2‐hydroxypropyl)‐β‐cyclodextrin as a chiral selector added to the mobile phase. For this approach, the enzymatic reaction was conducted under optimal conditions (pH 6, 30 °C), incubated for 30 min for complete conversion and afterwards extracted with ethyl acetate. For the standard measurements, d‐ or l‐PLA were added to the assays instead of the substrate PPA. HPLC analysis of the extract of the FrsC assay showed a peak at 10.97 min, which coincides with the standard measurement of l‐PLA (d‐PLA: 11.84 min, Figure 4A). It can thus be assumed that the reaction product of FrsC is l‐configured. To investigate which product is incorporated by the NRPS FrsE, the A‐PCP didomain of module 3 was cloned into pET28a, heterologously expressed in *E. coli* BL21 (DE3), purified and assayed with the mass spectrometry‐based *γ*‐^18^O_4_‐ATP exchange assay.^[37]^ In addition, an *E. coli* BL21 (DE3) strain co‐expressing FrsE A‐PCP3 and the MLP FrsB was constructed and the protein complex purified and assayed analogously (Figure S5). The results clearly show that FrsB is, as observed for FrsA A1 and FrsD A2,^[33]^ crucial for catalytic activity of FrsE A3, since significant substrate conversion was only detected in presence of the MLP (Figure 4B, Table S2). The assays demonstrated a rather low substrate specificity for FrsE A3. The highest exchange of heavy ATP was found with 52 % for l‐PLA, while the substrate d‐PLA showed 42 % conversion. These results thus suggest that FrsE A3 preferentially activates l‐PLA, although the difference is too insignificant to make a precise determination about *in vivo* activity. The alternative substrate l‐phenylalanine was converted to 48 %. Other structurally similar substrates like OH‐PLA and F‐PLA were not activated by FrsE A3, suggesting that structural modifications to the phenyl ring negatively affect A domain substrate binding.

Another experimental approach to study incorporation of a precursor into the assembly line is to perform *in vivo* feeding experiments. We therefore prepared a *C. vaccinii frsC* deletion mutant via a recently established knock‐out system.^[32]^ In doing so, the major part (822 bp) of *frsC* was replaced by the resistance cassette from pPS858 encoding gentamicin resistance and the green fluorescent protein (GFP) (Δ*frsC*::FRT).^[38]^ The respective genes were inserted in opposite direction regarding the other genes of the *frs* BGC to avoid any downstream effects. We then attempted to complement and restore FR biosynthesis by feeding either d‐ or l‐PLA to the Δ*frsC*::FRT mutant strain. The production of FR in the *C. vaccinii* WT and knock‐out strains was measured via HPLC‐MS analysis of butanolic extracts from the fermented strains. Analysis of the EICs for the proton adduct of FR (*m/z*: 1002.54) of the *C. vaccinii* Δ*frsC*::FRT feeding experiments (Figure 4C) showed a high production of FR in the *C. vaccinii* WT. In the *C. vaccinii* Δ*frsC*::FRT extracts, no FR could be detected, showing that the presence of FrsC is mandatory for FR biosynthesis. However, by the addition of l‐PLA to the mutant strain, FR production was restored to 3.6±0.14 %, compared to the WT, whereas feeding of d‐PLA to the mutant restored FR production by only 0.6±0.09 %.

Since the PLA‐incorporating A domain appears to be promiscuous (Figure 4B), these differences can be explained by the presence of a functional E domain (FrsE C4) downstream in module 3 (Figure S6),^[29]^ as d‐PLA would represent the false substrate for the E domain, leading to reduced incorporation. Taken together, the data obtained in this study thus confirm our hypothesis that l‐PLA is indeed the reaction product of FrsC, subsequently activated by the FrsE3A domain, incorporated into the growing FR peptide chain and finally epimerized to d‐PLA, present in the natural product (Figure 2).

**Figure 5:**
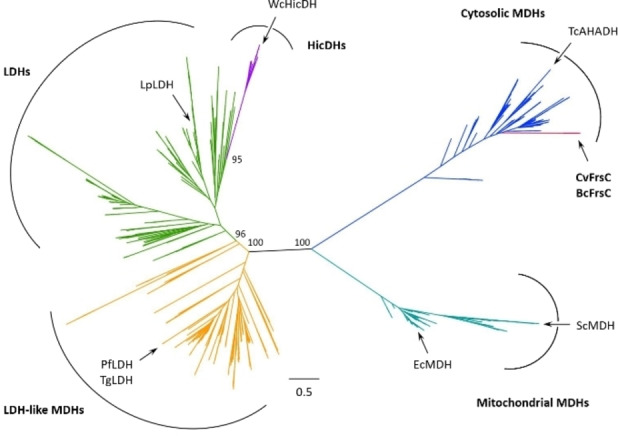
Phylogeny of FrsC. Besides FrsC, the tree represents 903 members of the l‐LDH/l‐MDH superfamily. The five major clades of this superfamily are indicated, as well as the position of different enzymes mentioned in this study. The tree branch consisting of CvFrsC and BcFrsC is highlighted in red. The scale bar represents 0.5 amino acid substitutions per site. The bootstrap (1000 replicons) values for certain nodes are given.

FrsC catalyzes an unconventional reaction in natural product biosynthesis. Therefore, we sought to bioinformatically investigate this enzyme in the context of other members of the LDH/MDH superfamily. We had already performed an alignment of FrsC with four other l‐LDHs and MDHs, revealing that FrsC contains different residues in the substrate binding pocket, which is indicative for an altered substrate specificity.^[29]^ To extend the bioinformatic analysis, we constructed a phylogenetic tree with both FrsC homologues (72 % identity) and 903 other l‐LDH/l‐MDH sequences, revealing the five distinct clades of mitochondrial and cytosolic‐like MDHs, LDH‐like MDHs, LDHs, and hydroxyisocaproate‐related dehydrogenases (HicDH), which together form this superfamily (Figure 5).^[39]^ Here, FrsC clusters with the group of cytosolic‐like MDHs. This is in accordance with earlier reports that the closest FrsC homologue is an MDH of *Deinococcus* sp. Leaf326.^[29]^ In support, our experiments showed that NADH, as well as NADPH, are both accepted as cofactors, a trait that is characteristic for l‐MDHs, as l‐LDHs were found to exclusively accept NADH as electron donor.^[40]^

**Figure 6:**
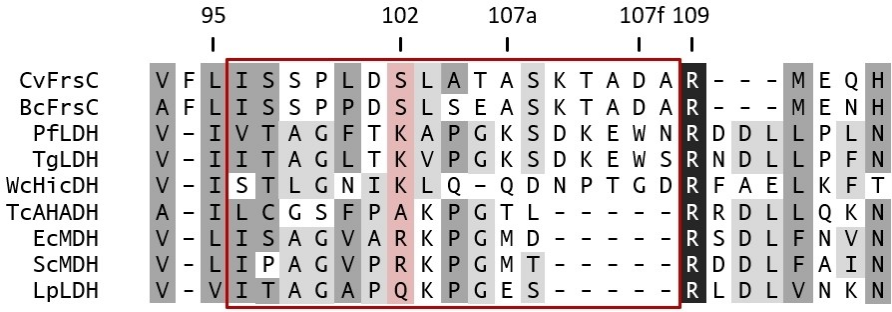
Alignment of the substrate specificity loop regions of both FrsC homologues and other enzymes of the l‐LDH/l‐MDH superfamiliy. The ‘specificity residue’ at position 102 is highlighted (light red) as well as the catalytic loop (red frame) as defined by Madern, 2002.^[40]^ Enzymes: PfLDH=*Plasmodium falciparum* LDH, TgLDH=*Toxoplasma gondii* LDH, WcHicDH=*Weissella confusa* HicDH, TcAHADH=*Trypanosoma cruzi* l‐alpha‐hydroxyacid dehydrogenase, EcMDH=*Escherichia coli* K‐12 MDH, ScMDH=*Saccharomyces cerevisiae* MDH, LpLDH=*Lactiplantibacillus plantarum* LDH. Amino acid similarities between all sequences are indicated as follows: black=100 % identity; dark grey=≥75 % similar, light grey=55–75 % similar. Full alignment in Figure S8.

While different enzymes within the LDH, MDH and HicDH clades also show the ability to reduce PPA to PLA,[^41^, ^42^, ^43^] the catalytic efficiency of this reaction is inferior compared to this of their native substrates. In contrast, FrsC shows activity exclusively with PPA or OH‐PPA as substrate (Figure 3D). Such a high specificity for an aromatic substrate hints at the evolution of a new function from a former MDH. As suggested before, this is most likely due to the unusual structure of the FrsC substrate specificity loop (Figure 6, Figure S7).^[29]^ This region contains the ‘specificity residue’ at position 102, which is Arg in case of l‐MDHs (e. g. EcMDH) and Gln in case of canonical l‐LDHs (e. g. LpLDH).^[44]^ Alterations within this region have been associated with functional changes of the respective enzymes.^[40]^ For example, the exchange of the ‘specificity residue’ to Lys as well as a loop insertion of five amino acids was essential for the evolution of apicomplexan LDHs (e. g. LDHs of *Plasmodium falciparum*, PfLDH, or *Toxoplasma gondii*, TgLDH) from a MDH ancestor. Residues in the apicomplexan insertion are numbered using the residue that precedes the insertion, K107, with letters added to each of the successive residues (K107a to W107f). In addition, due to the insertion Trp107f occupies the same space as Arg102 in MDHs, while Lys102 is excluded from the active site.^[39]^ HicDHs all contain an amino acid other than Gln at position 102, while their loop is extended by a varying number of amino acids (e. g. the HicDH of *Weissella confusa* (basonym: *Lactobacillus confusus*), WcHicDH; Figure 6).^[39]^

An alignment with selected LDHs and MDHs shows that FrsC, like PfLDH, TgLDH and HicDH possesses a five‐residue insertion in the catalytic loop (Figure 6, Figure S8). In FrsC, Asp107f aligns with Trp107f of PfLDH, which was shown to be involved in substrate recognition by PfLDH, also supported by structural model comparisons (Figure S7). This residue is thought to discriminate between pyruvate and oxaloacetate, either by a preferred interaction with the hydrophobic pyruvate C3 methyl group instead of the negatively charged oxaloacetate methylene carboxylate or by steric occlusion of the larger oxaloacetate.^[39]^ However, it is unlikely that Asp107f plays a similar role. First, interaction of the charged Asp107f with the hydrophobic phenyl residue of PPA is not favorable, second, in FrsC no occlusion of larger residues needs to take place. It is therefore more plausible that the extended loop of FrsC increases the active site space, thereby allowing the binding of bulkier substrates, as it is the case for WcHicDH.^[43]^ In this context, the relatively high number of Ala residues within the catalytic loop of FrsC is noteworthy, which might also contribute to enlarging the active site.

Interestingly, the *Trypanosoma cruzi* l‐alpha‐hydroxyacid dehydrogenase (TcAHADH), which belongs to the cytosolic‐like MDH clade and has been shown to utilize the aromatic p‐hydroxyphenyl pyruvate as substrate, shows no specificity loop insertion (Figure 6). However, here the ‘specificity residue’ is alanine and mutation of Ala102 to Arg was shown to re‐enable the reduction of oxaloacetate.[^45^, ^46^]

In an earlier analysis, we reported Asp101 as the ‘specificity residue’ for BcFrsC.^[29]^ In contrast, in this more detailed bioinformatic analysis, Ser102 takes this position. If one of both residues or an amino acid of the extended loop interacts with the substrate, thereby leading to an altered substrate affinity, however, can only be answered by further experimental analyses, such as structural or mutational studies.

Of note, the enzyme found to catalyze PLA formation in the CD natural product PF1022 belongs to the family of glycerate dehydrogenases (EC 1.1.1 29).^[47]^ This fungal enzyme, catalyzing the same reaction as FrsC, may thus represent a convergent case of evolutive adaptation of a primary metabolic enzyme for secondary metabolism, but from a different enzyme family. The comparatively low catalytic activity of FrsC supports the theory that enzymes involved in specialized metabolism exhibiting high specificity and reduced efficiency have evolved from primary metabolic enzymes that generally appear to have high turnover rates and rather low substrate specificity.^[48]^

## Conclusion

We have biochemically and bioinformatically characterized the enzyme FrsC from the biosynthetic pathway of the selective G protein inhibitor FR900359. FrsC reduces its substrate PPA, using the coenzyme NADPH as hydride donor, to l‐PLA which is subsequently activated and epimerized by the Frs NRPS assembly line. From our experimental and bioinformatic results we conclude that FrsC has evolved from an MDH, involved in primary metabolism to a highly specific phenylpyruvate reductase, involved in specialized metabolism of a bioactive natural product.

## Experimental Section

**Cell culture**: If not stated otherwise, liquid cultures of all bacteria were grown in LB medium supplemented with the appropriate antibiotic. The *E. coli* strains were cultivated at 220 rpm and 37 °C, whereas the strains *C. vaccinii* MWU205 (DSMZ 25150) and *C. vaccinii* Δ*frsC*::FRT were incubated at 220 rpm and 30 °C. Precultures (5 ml) of the *C. vaccinii* strains were inoculated from single clones grown at 30 °C on LB agar, while *E. coli* precultures were inoculated from cryo‐cultures stored at −80 °C.

**Cloning and transformation**: Amplification of *frsC* (amplified with the primers ‘*Cv*_*frsC‐His6‐N_for‐Bam*HI, TATGGATCCATGAAAAATTCCGT CCG’ and ‘*Cv*_*frsC‐His6‐N_rev‐Xho*I, GTTACTCGAGTTACAACAAATTG AATTGC’), *frsE3 A+PCP* (amplified with the primers ‘Chv_FrsE3_A_for‐*Hin*dIII, TATAAGCTTTGGACGAGCGCCGGCAGG’ and ‘Chv_FrsE3 _PCP_rev‐*Xho*I, TATCTCGAGTCACACCCGCTCGTTCCGGTC’) and *frsB* (amplified with the primers ‘FrsB_for, GCGCATATGAGC AATCCCTTTGATGAT’ and ‘FrsB_rev, GCGTTAATTAATTATTTAT CATCGCACTCCAT’) was performed via Q5 (New England Biolabs) PCR using genomic DNA of *C. vaccinii* as template. The resulting inserts were then cloned into pET‐28a(+) (FrsB into pCDFDuet‐1) using the restriction sited indicated (underlined) in the primers (*frsC*: *Bam*HI and *Xho*I; *frsE3A‐PCP*:*Hind*III and *Xho*I; *frsB*: *Nde*I and *Pac*I). The vector pET28a::*frsC‐N‐His* was transformed into competent *E. coli* XL1‐Blue (Stratagene). The vectors pET28a::*frsE3_A‐PCP* and pCDFDuet‐1::*frsB* were transformed into competent *E. coli* alpha‐Select Silver (New England Biolabs) using heatshock transformation. After selection by addition of the appropriate antibiotic (pCDFDuet1: apramycin; pET28a: kanamycin), the constructs were verified by Sanger DNA sequencing and the plasmids re‐isolated and transformed into the expression strain *E. coli* BL21 (DE3) (New England Biolabs) via heat shock transformation. For co‐expression of FrsB and FrsE A‐PCP3, the vectors pET28a::*frsE3_A‐PCP* and pCDFDuet‐1::*frsB* were co‐transformed.

**Protein expression**: Overproduction of FrsC−N‐His was performed in *E. coli* BL21 (DE3) carrying the vector pET28a::*frsC*‐N‐His. For the overproduction of FrsE3A‐PCP, two approaches were performed: One in the presence of FrsB in *E. coli*, carrying the vectors pET28a:: *frsE* A‐PCP N‐His and pCDFDuet‐1::*frsB*, and another with an *E. coli* strain carrying only pET28a *Chv frsE* A‐PCP N‐His. In a conical flask, 200 ml TB medium supplemented with the appropriate antibiotic was inoculated (1 : 100) with a fresh overnight culture. The main culture was incubated at 37 °C and 220 rpm until an OD_600_ of 1.0. After 30 min incubation on ice, protein expression was induced with 0.4 mM IPTG and the cultures were incubated for 16 h at 16 °C and 220 rpm.

**Protein purification**: The cells were pelleted (15 min, 4,000 rpm, 4 °C) and resuspended in 2.5 ml lysis buffer (50 mM NaH_2_PO_4_, 300 mM NaCl, 10 mM imidazole, pH 8.0) per g pellet. The cells were further processed under continuous cooling. Cell lysis was performed by sonification (10×10 pulses at 20 % initial strength) and the lysate was centrifuged (10 min, 10,000 rpm, 4 °C. The supernatant was transferred to a new centrifuge tube. 1 ml nickel‐nitrilotriacetic acid (Ni‐NTA) was added and incubated (1 h, 120 rpm, 4 °C). The mixture was purified over a polypropylene column (Qiagen) with two washing steps with 4 ml each of wash buffer I (50 mM NaH_2_PO_4_, 300 mM NaCl, 20 mM imidazole, pH 8.0) and wash buffer II (50 mM NaH_2_PO_4_, 300 mM NaCl, 35 mM imidazole, pH 8.0). The protein was eluted with 2.5 ml of elution buffer (50 mM NaH_2_PO_4_, 300 mM NaCl, 250 mM imidazole, pH 8.0). Exchange of the buffer system to Tris buffer (20 mM Tris pH 7.5) was performed with PD‐10 columns (GE Healthcare) following the manufacturers gravity protocol. The rebuffered protein was transferred to an Amicon® Ultra‐4 centrifuge filter unit. The protein was brought to the desired concentration by repeated centrifugation (5 min, 7,500 g, 4 °C).

**His‐Tag removal**: FrsC was cloned into pHis8‐TEV (kanamycin resistance) (FrsC‐TEV‐His). The TEV protease (expressed from the pTEV, kanamycin resistance) and FrsC‐TEV‐His were purified as described above, mixed in a 1 : 5 ratio (m/m) and incubated overnight at 4 °C. The next day 1 ml Ni‐NTA was added and the mixture was incubated for 1 h. To remove the undigested protein as well as the protease, the mixture was purified over a propylene column and washed with 10 ml wash buffer I. The flowthrough and the wash fraction contained FrsC without His‐tag and were collected, pooled and concentrated.

**FrsC activity assay**: The assay solution comprised a volume of 1 ml and was composed of 100 mM buffer, 2 mM substrate (phenylpyruvate), 0.5 mM coenzyme (NADPH) and 1 μM FrsC. Substrate and buffer were preincubated at the desired temperature for 30 min. Buffer and substrate were transferred into the cuvette. The coenzyme was added, the zero value was determined and after baseline configuration FrsC was added to the mixture. For 10 min, the absorbance at 340 nm was measured every 5 s. Using the graphical representation and the Lambert Beer's law, the activity of FrsC was calculated. To determine the optimal reaction temperature, pre‐incubation temperatures were varied from 20–80 °C. For the identification of the optimal pH, buffers were varied. For pH values of 3–5, a citric acid phosphate buffer (0.1 M C_6_H_8_O_7_×H_2_O, 0.2 M Na_2_HPO_4_×2 H_2_O, pH 3.0–5.0), for the neutral pH range, sodium dihydrogen/disodium hydrogen phosphate buffer (0.1 M NaH_2_PO_4_×2 H_2_O, 0.1 M Na_2_HPO_4_, pH 6.0–8.0) and for basic pH values of 9–10, sodium carbonate/sodium hydrogen carbonate buffer (0.1 M Na_2_CO_3_, 0.1 M NaHCO_3_, pH 9.0‐10.0) was used. To determine the kinetic parameters, phenylpyruvic acid concentration was varied between 0.1 mM–7 mM. The results were visualized with GraphPad Prism in a Michalis‐Menten‐curve and a Hanes‐Woolf plot, which allows the determination of *K*_M_ and *k*_cat_. Assays were performed in triplicates. To determine FrsC substrate specificity, 2 mM of 4 OH‐phenylpyruvic acid, pyruvic acid, phenyllactic acid, malate, l‐phenylalanine, l‐tyrosine, or phenylglyoxylate were added to the reaction under optimal conditions.

**Chiral HPLC**: In order to extract the reaction product of FrsC for subsequent determination of its configuration, the procedure was similar to the activity assay. The reaction mixture contained 3 mM purified FrsC, 2 mM NADPH and 1 mM PPA and was incubated at optimal reaction conditions (see above). For the standard measurements, 1 mM d‐ or l‐PLA were added to the reaction instead of PPA. After 30 min, the reaction was quenched by addition of HCl, thus lowering the pH value to 2. The same volume of EtOAc was added and shaken. After a phase separation, the organic phase was isolated, vacuum dried and stored at ‐20 °C. For HPLC analysis, the extracts were dissolved in 1 ml MeOH and measured on an HP XBridge® BEH Shield RP18 Column, 130 Å, 3.5 μm, 4.6 mm×100 mm from Waters as stationary phase. The mobile phase was aqueous 0.05 % formic acid buffer (pH 2.5) containing 10 % methanol and 10 mM Hp‐β‐cyclodextrin. The flow rate was 0.5 ml/min over 40 min measuring time at a pressure of about 150 mbar. 15 μL of samples were injected.

**γ^18^O_4_ ATP exchange assay**:^[37]^ To investigate substrate specificity of FrsE3A, the activation of FrsE3 A‐PCP was tested in presence or in absence of FrsB. The PPi‐ATP exchange took place in a mixture of 3 mM (amino) acid, 15 mM PPi, 3 mM γ‐^18^O_4_‐ATP, 15 mM MgCl_2_ and 5 μM of the purified enzyme in 6 μl Tris buffer (20 mM, pH 7.5). After 1.5 h at 22 °C, the reaction was stopped by the addition of 6 μl of 10 mg/ml 9‐aminoacridine in acetone. The tested acids were PPA, l‐PLA, d‐PLA, l **‐**Phe, OH‐PLA, F‐PLA. Measurements were performed in triplicate. MALDI‐TOF‐MS analyses were performed on a Bruker Autoflex III (Bruker Daltonik GmbH, Bremen). For the measurement, 1 μL of the analyte/matrix mixture was spotted to the ground steel target and dried at room temperature. The measurement was performed in negative mode in a *m/z* range of 300–1000. In a random walk with 300 shots each, 3000 spectra were summed up. A Bruker peptide mix in negative mode was used as calibrant (*m/z* range 1000–3000, HCCA as matrix). The software FlexControl 3.3 was used for analysis and FlexAnalysis 3.3 for data processing.

**Construction of** ***C. vaccinii*** **Δ*frsC*::FRT**: The *frsC* deletion mutant of *C. vaccinii* MWU205 was generated following the protocol described elsewhere.^[32]^ In general, a sequential cloning of the *frsC* upstream (C‐up; amplified with the primers ‘SphI‐frsC‐up_for, AGTGCATGCGGCGATTTG CTGCTATTTCG’ and ‘SalI‐frsC‐up_rev, AGAGTCGACAGAAATAGCTA CACGGACGG’) and downstream (C‐dn; amplified with the primers ‘BglII‐frsC‐dn_for, TGAAGATCTGGTTTTCCAGTTGTAGCCG’ and ‘SacI‐frsC‐dn_rev, AACGAGCTCTGAAATCAGGACTCCAGTCC’) regions and the GemR resistance cassette (FRT; amplified with the primers ‘BamHI‐FRT_for, TGTGGATCCAGCTTCAAAAGCGCTCTGA’ and ‘SalI‐FRT_rev, TGTGTCGACGGGGATCTTGAAGTTCCT’) into pUC19 (cloning order: FRT > C‐up > C‐dn; employed restriction enzymes are indicated in the respective primer names) was performed. The resulting insert ‘C‐up_FRT_C‐dn’ was then subcloned into pEX18Tc using *Sac*I and *Sph*I.^[38]^ Afterwards, triparental mating of *E. coli* NEB Turbo (New England Biolabs) carrying the resulting vector pEX18Tc::Δ*frsC* with *E. coli* ET12567 pUB307 and *C. vaccinii* MWU205, as well as appropriate selection yielded the strain *C. vaccinii* MWU205 Δ*frsC*::FRT.

**Feeding experiments**: For the analysis of the untreated *C. vaccinii* MWU 205 (WT) and *C. vaccinii* Δ*frsC*::FRT strains, a 100 ml conical flask containing 50 ml LB medium with 200 μg/mL ampicillin was inoculated in a 1 : 100 ratio with a fresh overnight culture. For the feeding experiments, 500 μl of a preculture were added to 45 ml LB medium. The substrates to be fed (d‐ and l‐PLA) were dissolved in 5 ml LB medium and then added to a final concentration of 3.6 mM. The cultures were incubated (36 h, 30 °C, 220 rpm) and subsequently, 40 ml *n*‐butanol were added for extraction (24 h, 30 °C, 200 rpm). Afterwards, the cultures were centrifuged (20 min, 4,000 rpm). The organic phase was evaporated and the extracts stored at −20 °C until measurement. Feeding experiments were performed in triplicate.

**LC/MS**: The samples were dissolved in methanol to a concentration of 1 mg/ml crude extract and analyzed by HPLC‐MS with a micrOTOF‐QIII mass spectrometer (Bruker) with ESI source coupled with a HPLC Dionex Ultimate 3000 (Thermo Scientific) using a Waters Atlantis T3, 5 μm, 4.6×50 mm column. The column temperature was 25 °C. MS data were acquired over a range from 100–3000 m/z in positive mode. Elution started with a flow of 0.3 ml/min at 90 % H_2_O containing 0.1 % acetic acid (A) and 10 % acetonitrile containing 0.1 % acetic acid (B). After 1 min, a linear gradient to 100 % B was run for 9 min, followed by an isocratic step with 100 % B for 4 min. 5 μl of a 1 mg/ml sample solution were injected per run.

**Phylogenetic tree**: The majority of sequences for the construction of the evolutionary tree were taken from the InterPro database. For this, 871 sequences of the l‐lactate/malate dehydrogenase family marked as reviewed by UniProt and with a length of more than 270 amino acids were downloaded from the InterPro database (reference no. IPR001557). As the group of HicDHs was underrepresented in this dataset, further homologues of WcHicDH (*Weissella confusa* HicDH, P14295) were extracted from the non‐redundant protein database via NCBI BLAST. From the top 100 hits, two representatives per species were chosen. This yielded 28 additional protein sequences, which were added to the protein list. In addition, the enzymes used for the protein alignment of FrsC with other members of the l‐LDH/MDH superfamily, which were not already present in the dataset, were added.

The resulting 904 protein sequences were aligned using the ‘Pairwise/Multiple Align’ function of Geneious Pro 5.6.7 (MUSCLE alignment; default settings) and the resulting file submitted to IQ‐TREE 1.6.12 for phylogenetic tree construction. To identify the best fitting model for our dataset, the model selection function (ModelFinder Plus) was used. A maximum‐likelihood tree was then constructed with the proposed ‘LG+R10’ model and an ultrafast bootstrap approximation (1000 bootstrap replicates).[^49^, ^50^, ^51^] The final tree was visualized with FigTree 1.4.4 (http://tree.bio.ed.ac.uk/software/figtree/).

**Alignment and model**: The sequences of CvFrsC (*Chromobacterium vaccinii* MWU205; accession no. QPI18725), PfLDH (*Plasmodium falciparum* LDH; Q27743), TgLDH (*Toxoplasma gondii* LDH; Q27797), WcHicDH (*Weissella confusa* HicDH, P14295), TcAHADH (*Trypanosoma cruzi* l‐alpha‐hydroxyacid dehydrogenase; AAF36775), EcMDH (*Escherichia coli* K‐12 MDH, P61889), ScMDH (*Saccharomyces cerevisiae* MDH; NP_012838), LpLDH (*Lactiplantibacillus plantarum* LDH; ACN66626) were aligned using the ‘Pairwise/Multiple Align’ function of Geneious Pro 5.6.7 (Geneious alignment; Settings: Cost matrix ‘Blosum80’, Gap open penalty ‘12’, Gap extension penalty ‘3’, Alignment type ‘Global alignment’, Build guide tree ‘Yes’, Refinement iterations ‘2’). Afterwards, the sequence of BcFrsC (“*Ca*. Burkholderia crenata”; KNE75169) was added by using the ‘Multiple align’ function (‘Profile align’ using the same settings as above). The homology model of CvFrsC was generated by I‐TASSER, basing on the model of the l‐lactate dehydrogenase of *Plasmodium falciparum* (SMTL ID: 3zh2.1) and visualized with PyMOL (Version 2.5.1).

## Conflict of interest

The authors declare no conflict of interest.

1

## Biographical Information

*Max Crüsemann is currently a Junior Research Group Leader in the Institute of Pharmaceutical Biology, University of Bonn. He studied Pharmacy at the University of Marburg, and obtained his PhD from the University of Bonn for biosynthetic studies on a complex natural product (advisor Prof. Jörn Piel). After postdoctoral training on natural product genomics and metabolomics in the group of Prof. Bradley Moore at the Scripps Institution of Oceanography, San Diego, he returned to Bonn to Prof. Gabriele König‘s group and started his independent career in 2017. He is interested in omics‐guided discovery and biosynthetic studies of bacterial natural products*.

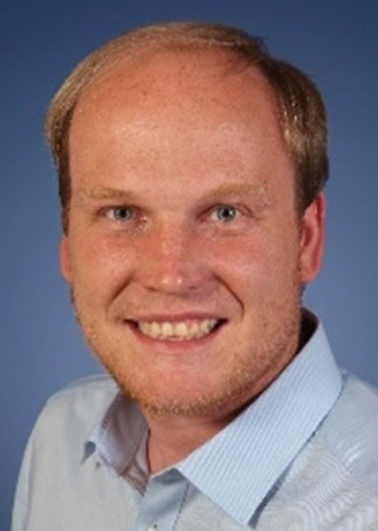

## Supporting information

As a service to our authors and readers, this journal provides supporting information supplied by the authors. Such materials are peer reviewed and may be re‐organized for online delivery, but are not copy‐edited or typeset. Technical support issues arising from supporting information (other than missing files) should be addressed to the authors.

Supporting Information

## Data Availability

The data that support the findings of this study are available from the corresponding author upon request.
