# Supplementary material for: A Specialized Dehydrogenase Provides l‐Phenyllactate for FR900359 Biosynthesis
Source: Chembiochem. 2021 Dec 9;23(10):e202100569. doi: 10.1002/cbic.202100569 (PMC9299796; doi:10.1002/cbic.202100569)
Supplement: Supplementary file 1 — Supporting Information [file CBIC-23-0-s001.pdf]

ChemBioChem

Supporting Information

A Specialized Dehydrogenase Provides L-Phenyllactate for FR900359 Biosynthesis

Sophie Klöppel[+], René Richarz[+], Daniel A. Wirtz, Natalia Vasenda, Gabriele M. König, and Max Crüsemann*

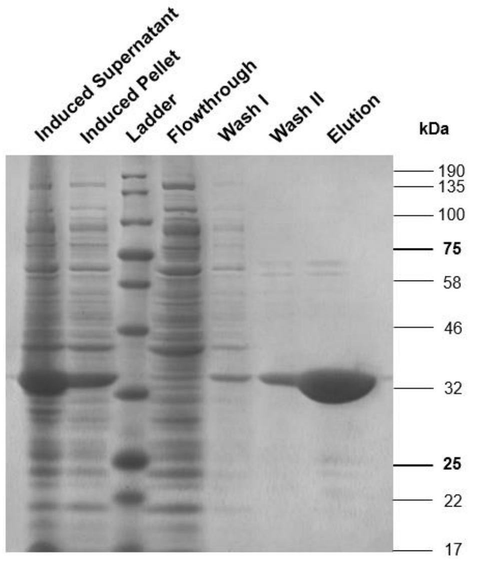

Figure S1: SDS-PAGE gel of FrsC purification (35.4 KDa) after expression in *E. coli* BL21(DE3).

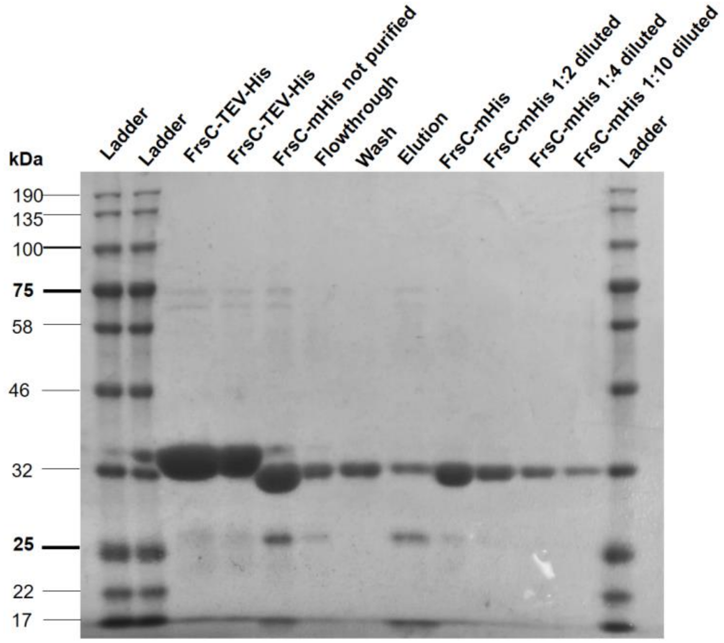

Figure S2: SDS-PAGE gel of FrsC purification after removal of the histidine tag.

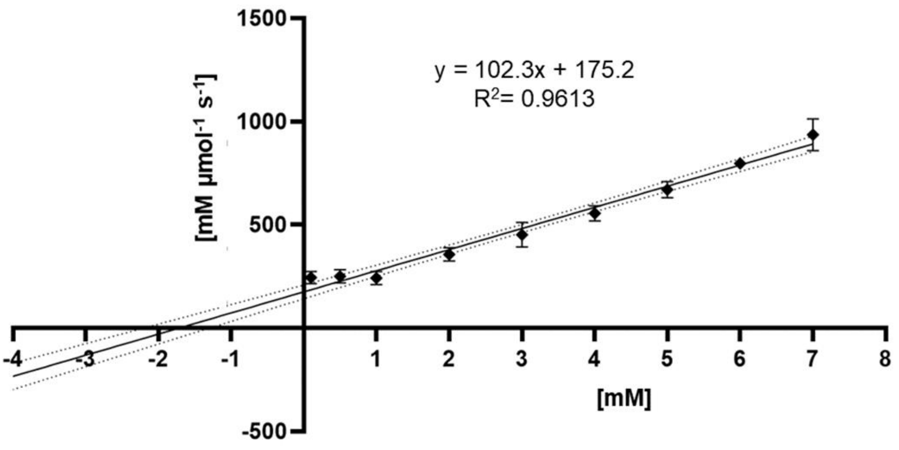

Figure S3: Hanes-Woolf plot for calculation of FrsC kinetic parameters. Linear equation and coefficient of determination are shown in the graph (dashed lines = 95 % confidence interval).

Table S1: **Tabular presentation of the kinetic parameters calculated based on the graph from Figure S3 and from the Michaelis-Menten curve (Figure 3 B).**

	V_{max} [µmol min^{-1} mg^{-1}]	K_m [mM]	k_{cat} [s^{-1}]	k_{cat}/K_m [mM^{-1} s^{-1}]
**Hanes-Woolf plot**	16.58 ± 1.47	1.71 ± 0.48	9.78 ± 0.87	5709.28 ± 2121.01
**Michaelis-Menten**	15.93 ± 1.34	1.37 ± 0.41	9.40 ± 0.79	6883.52 ± 2639.18

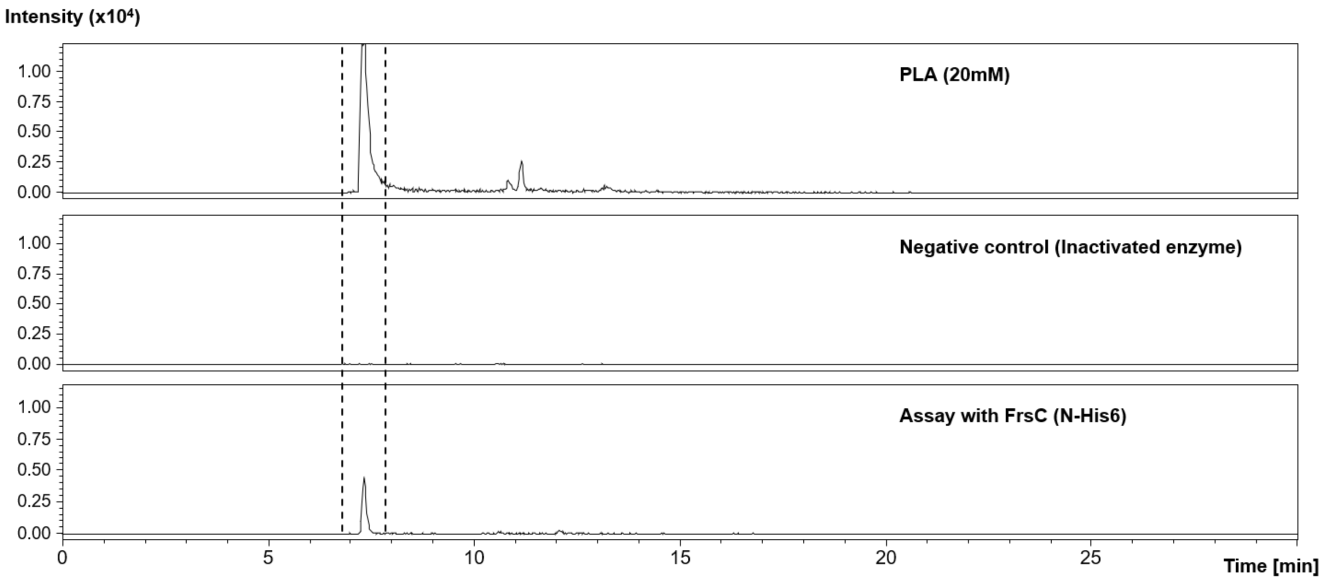

Figure S4: HPLC-MS chromatograms of FrsC assay, negative control and PLA standard.

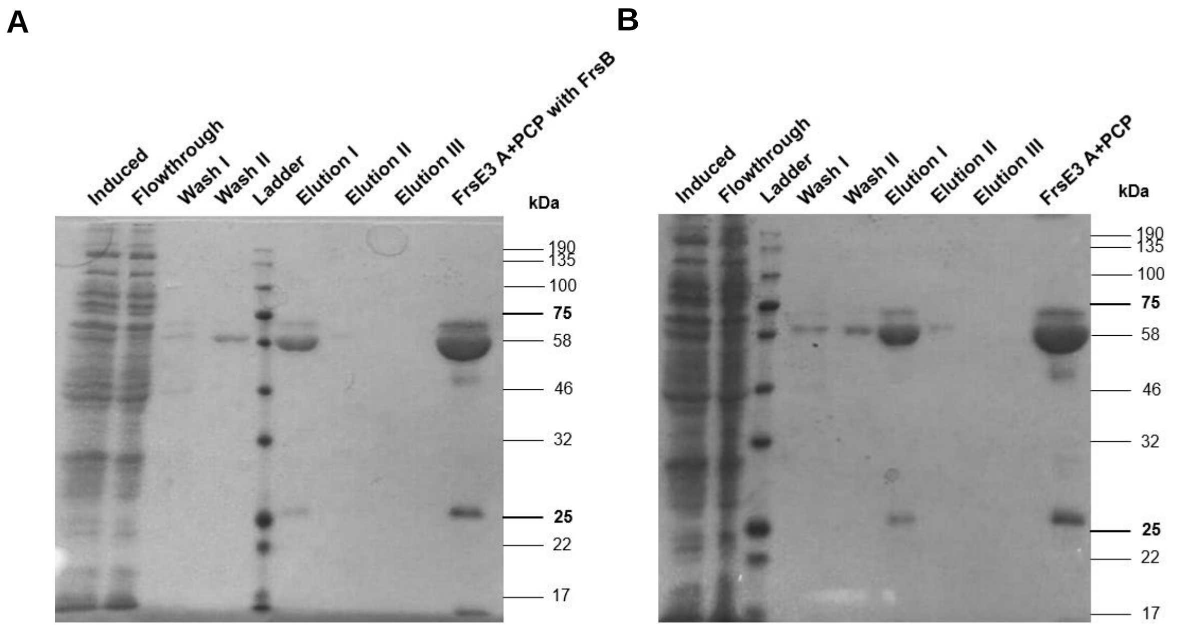

Figure S5: SDS-PAGE gel A) FrsE3A-PCP (69.5 KDa) coexpressed with FrsB (8.2 KDa), B) FrsE3A-PCP. Both were overexpressed and purified from *E. coli* BL21 (DE3).

Table S2: **Results of the $^{18}O_4$-ATP exchange adenylation assay for FrsE3 A-PCP with and without coexpression of FrsB**. Data are presented as mean values with SD. The assays were performed in triplicate.

		**Exchange [%]**	**Standard deviation [%]**
**FrsE3 A-PCP / FrsB**	Negative control	3.84	1.73
	PPA	2.45	1.47
	L-PLA	52.27	4.73
	D-PLA	42.19	3.64
	L-Phenylalanine	47.98	4.38
	OH-PLA	3.17	0.74
	F-PLA	8.04	0.24
**FrsE3 A-PCP**	Negative control	2.81	0.07
	PPA	2.20	0.04
	L-PLA	3.12	0.09
	D-PLA	8.90	0.81
	L-Phenylalanine	2.11	0.30

```
                                          E1
FrsE_C4      --GDLTLTPILRWMWENGPYR-----LFHQSQLLRAPSGLRRADLLAMVQALLDHHDALR   53
MycC_E       IKGPALLTPIQHWFFDQRYPD---LHHYNQAVMLYWKEGLNVPMLREVMRKIVEHHDALR   57
SrfA_E       VEGEVILTPIQRWFFEKNFTN---KHHWNQSVMLHAKKGFDPERVEKTLQALIEHHDALR   57
GrsA_E       VEGEIGLTPIQHWFFEQQFTN---MHHWNQSYMLYRPNGFDKEILLRVFNKIVEHHDALR   57
TycA_E       IAGNVPLTPIQKWFFGKNFTN---TGHWNQSSVLYRPEGFDPKVIQSVMDKIIEHHDALR   57
FrsE_C3      --EVLPLSYAQRRLWFTHCIKG-PSPAYNIPLMLRLAGKPAKEALQAALQDVLTRHESLR   57
FrsE_C5      -EEVLPLSPLQKGLLFHGLYDPAGVDPYVERLIYALEGELDAGALKQAVHGLLLQHSNLR   59
             *:   : :             :    :            :   .  :: :*. **

FrsE_C4      MRLHE-DDGEARMTILPVGTTRAEDCVRRIEIVGVDA--VERQVVLARE-TDEAILRLDS   109
MycC_E       MVYVP-AKHGYEARNREI----DEGDLFSLEVFSLLEE-NNVAQTIETL-SNEIQQSIQL   110
SrfA_E       MVYRE-GQEDVIQYNRGL-----EAASAQLEVIQIEGQAADYEDRIERE-AERLQSSIDL   110
GrsA_E       MIYKH-HNGKIVQINRGL-----EGTLFDFYTFDLTAN-DNEQQVICEE-SARLQNSINL   109
TycA_E       MVYQH-ENGNVVQHNRGL-----GGQLYDFFSYNLTAQ-PDVQQAIEAE-TQRLHSSMNL   109
FrsE_C3      TLCVEADDGEPMQHILPAQ----AVTVFRLETHVA-AS-VAEQS--EAV-VEASRHCFDL   108
FrsE_C5      ACFVDLGRGQPVQVIVPLS----ALPWQEIDLSML-GE-DEQQAVLEQMQEEDRHQRFDL   113
                         :                            ::

                                   ______E2______
FrsE_C4      ECGRLVQVVWLDAGSEEGWLRLVIHHLAVDGVSWRVLLSDWQQAWADVCVGCAISLDPVG   169
MycC_E       AEGPLIKLGLFQC-QDGDHLLIVAHHLVIDGVSWRILIEDIAAAYEQLLNGEAIQLPKKT   169
SrfA_E       QEGGLLKAGLFQA-EDGDHLLLAIHHLVVDGVSWRILLEDFAAVYTQLEQGNEPVLPQKT   169
GrsA_E       EVGPLVKIALFHT-QNGDHLFMAIHHLVVDGISWRILFEDLATAYEQAMHQQTIALPEKT   168
TycA_E       QEGPLVKVALFQT-LHGDHLFLAIHHLVVDGISWRILFEDLATGYAQALAGQAISLPEKT   168
FrsE_C3      STEIPLRATLFLAEGAPPLLLLLLHHIAADGDSLPVLAKNLEFAYLARHESRPPEWSLLA   168
FrsE_C5      SHAPLLSFVLIRLAVDRHRLIMSNHHILLDGWSGPLLWRELMKLYRSGGDLRAI---PRV   170
                   :    :         * :  **:  ** *  :*  :    :

FrsE_C4      TSFRNWALCLQRDAQSP-----QREAELAYWCSMLSTTDMPL-GRRAFDPARDTTRTKQS   223
MycC_E       DSYLLWAEQLKRYAESP-----EFEMKNQYWFQHEHIP-LPK-LPKDNEQEIGLAEDRET   222
SrfA_E       HSFAEYAERLQDFANSK-----AFLKEKEYWRQLEEQAVAAK-LPKDRESGDQRMKHTKT   223
GrsA_E       DSFKDWSIELEKYANSE-----LFLEEAEYWHHLNYYTENVQ-IKKDYVTMNNKQKNIRY   222
TycA_E       DSFQSWSQWLQEYANEA-----DLLSEIPYWESLESQAKNVS-LPKDYEVTDCKQKSVRN   222
FrsE_C3      VQYADYTLWQREWLGNIGTADSPAAHQLRYWRGALRGMPQVMALPTDRPRPPVATHRGGK   228
FrsE_C5      TPYRDYLDWLA----RR-----DLEPDRMAWRGYLRDLVTPTLLAPAAPT---EYVIQET   218
                         :  :                         .    *
                ___E3___                                E4
FrsE_C4      LSLSLPVRTTQALLTQAATRFHAQANDVLLTVFVLAMAAWRRQCMGHAPDALLFDLEGHG   283
MycC_E       IIVQWTAEETERLLKNAHRAYTTEMNDLLLTGLGIAIHRWTGH------EDILIHLEGHG   276
SrfA_E       IEFSLTAEETEQLTTKVHEAYHTEMNDILLTAFGLAMKEWTGQ------DRVSVHLEGHG   277
GrsA_E       VGMELTIEETEKLLKNVNKAYRTEINDILLTALGFALKEWADI------DKIVINLEGHG   276
TycA_E       MRIRLHPEETEQLLKHANQAYQTEINDLLLAALGLAFAEWSKL------AQIVIHLEGHG   276
FrsE_C3      VPFALPAAAHARLKTL-AETEAVTLSMVLQAGLSALLY-----RLGAG-SDVVIGGLLAG   281
FrsE_C5      YERALPDALASGLTAL-AEQLGVTLNTVIQGAWGRVLG-----CLTTS-QDVMFGSNVAG   271
                *          .  . ::        :              : .      *
                  _____E5_____                                        ___
FrsE_C4      RETQDTAIDLSRTVGWFTSLFPVRVRLDAVDLDDALGEGASLGRLLKSVKEQLHA-LPDR   342
MycC_E       RESIIPDLDISRTVGWFTSQYPVFLPIKA---------DHDISQRIKTVKEHLRK-IPQK   326
SrfA_E       REEIIEDLTISRTVGWFTSMYPMVLDMKH---------ADDLGYQLKQMKEDIRH-VPNK   327
GrsA_E       REEILEQMNIARTVGWFTSQYPVVLDMQK---------SDDLSYQIKLMKENLRR-IPNK   326
TycA_E       REDIIEQANVARTVGWFTSQYPVLLDLKQ---------TAPLSDYIKLTKENMRK-IPRK   326
FrsE_C3      RNDE----ALKDLIGFFVNAWVLRTDLSG---------HPDFHVLLRRVREQALQAYSHP   328
FrsE_C5      RPAEL--NGIEDMIGLFINTIPLRVRWSR---------GESIGDVLKRIQSEQVDLLEHQ   320
              *        :   :* * .   :    .             :   ::  :..
                E6                                  _____E7_____
FrsE_C4      GLGFGLLRYLNQGT-AAELAAHGQPQIGF------NYLGRFAASEGGDWQLASDV--GIE   393
MycC_E       GIGYGIIKYLS--DHREDREFTGQPEISF------NFLGQFDQDLQNGSIEVSPYSSGKI   378
SrfA_E       GVGYGILRYLTAPEHKEDVAFSIQPDVSF------NYLGQFDEMSDAGLFTRSELPSGQS   381
GrsA_E       GIGYEIFKYLTTEYLRPVLPFTLKPEINF------NYLGQFDTDVKTELFTRSPYSMGNS   380
TycA_E       GIGYDILKHVTLPENRGSLSFRVQPEVTF------NYLGQFDADMRTELFTRSPYSGGNT   380
FrsE_C3      DLPFEWLVEQLN----PIRSTSYHPLFQVVLVLQNNQRAR---FRLGGLVVEQQVLGTGT   381
FrsE_C5      YLDLVEIQ----------SQASHRDLFDSVYAFENYPVHANDEDEASGPRV-KVVSGGST   369
              :    :                   :  .                         .

FrsE_C4      AGQDP--EMPLPHPLSFDAHT--LDRTHGPELTAIWSWGSELFSSDEIAELAQLWQQAAM   449
MycC_E       A--------SDKHPLTYALDI--NGMISNGRLSLAISYCGKQYHKETMETCADLLKSSLR   428
SrfA_E       L--------SPETEKPNALDV--VGYIENGKLTMSLAYHSLEFHEKTVQTFSDSFKAHLL   431
GrsA_E       LGPDGKNNLSPEGESYFVLNI--NGFIEEGKLHITFSYNEQQYKEDTIQQLSRSYKQHLL   438
TycA_E       LGADGKNNLSPESEVYTALNI--TGLIEGGELVLTFSYSSEQYREESIQQLSQSYQKHLL   438
FrsE_C3      A----------KFDLAFNLFETMSDDGHPLGVTGDLEYACDLFDPPSAVRLAYRLSRLLE   431
FrsE_C5      T----------HYPLGLIVN-------PQAGLSLLFSYRPDCYRRCDIERIAAYLQCVLE   412
                           :     :    :         :    .

          FrsE_C4      ALAEHVTRPGAGGRTPSDLPLVHLHQAQIEQL          481
          MycC_E       QVIEHCTAQDQVQLTPSDISLKEISIDELDQF          460
          SrfA_E       RIIEHCLSQDGTELTPSDLGDDDLTLDELDKL          463
          GrsA_E       AIIEHCVQKEDTELTPSDFSFKELELEEMDDI          470
          TycA_E       AIIAHCTEKKEVERTPSDFSVKGLQMEEMDDI          470
          FrsE_C3      IWSAAPSQSIAIL-------------------          444
          FrsE_C5      AFAVDSTQPIAQL-------------------          425
```

Figure S6. Multiple sequence alignment (Clustal Omega 1.2.4) of different epimerization (E) domains with the proposed condensation (C) domains of FrsE (FrsE_C3, FrsE_C4, FrsE_C5). The highly conserved E domain core motifs (E1 – E7) as reported by Marahiel et al. (*Chem. Rev*.1997, 97, 2651) are shown in yellow. The corresponding regions of the C domain FrsE_C3/5 sequences are also highlighted in yellow to allow comparison MycC_E: E domain of Mycosubtilin synthase C (accession: Q9R9I9), SrfA_E: E domain of Surfactin synthase A (accession: P27206), GrsA_E: E domain of Gramicidin S synthase A (accession: P0C061) TycA_E: E domain of Tyrocidine synthase A (accession: P09095).

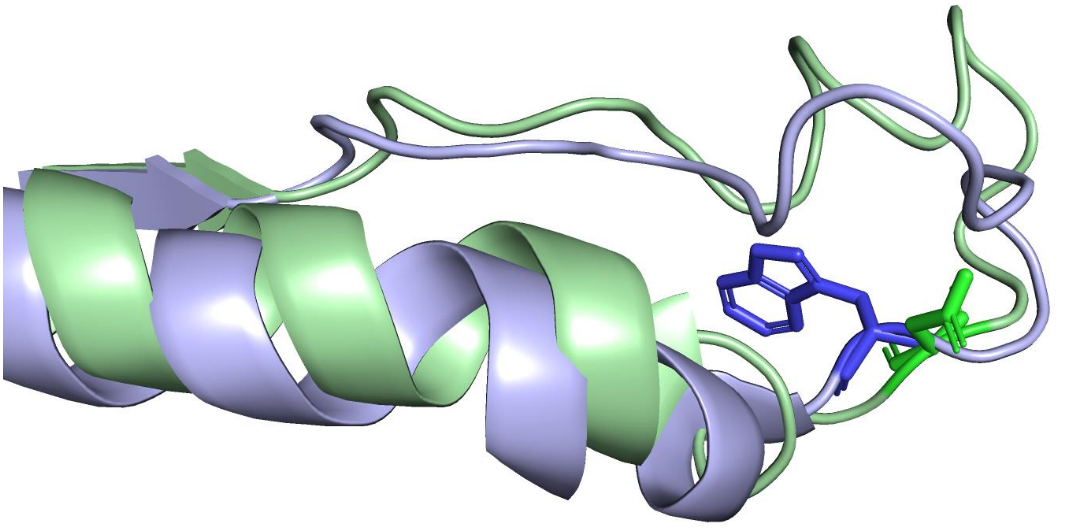

Figure S7. Alignment via PyMol of the structural models of FrsC of *C. vaccinii* (light green) and the LDH of *Plasmodium falciparum* (light blue) with focus on the substrate loop. FrsC was modelled using I-TASSER (08/2021). It was assumed that the specificity residue is the amino acid at position 102. In the case of FrsC, this is an Asp (green), whereas a Trp (blue) is located at a comparable position (107f) in the LDH of *P. falciparum*.

```
  CvFrsC   M---------KNSV---R-VAISGAAGRIANNLLFSIASGQLLGDQQPISLSLLETPARLSMLNGIAMELHDGVFPLLAGVEVS---DDPWQAFEGADY
  BcFrsC   M-------------TTPVRVVISGAVGRIGNSLLFNIASGQLLGSEQPVVLSLLEASSRLPMLSGVLMELHDGAFPLLANVEIH---DDPWLAFDGADY
   PfLDH   M---------APKA----KIVLVGS-GMIGGVMATLI-VQKNLGDV-----VLFDIVKN--MPHGKALDTSH--TNVMAYSNCKVSGSNTYDDLAGADV
   TgLDH   M---------TGTVSRRKKIAMIGS-GMIGGTMGY-LCVLRELADV-----VLFDVVTG--MPEGKALD--DSQATSIADTNVSVTSANQYEKIAGSDV
 WcHicDH   M---------------ARKIGIIGL-GNVGAAVAHGL-IAQGVADD----YVFIDANEA--KVKADQIDFQDAMANLEAHGNIVI---NDWAALADADV
 TcAHADH   MF-------FEGACA---KVVVSGAAGQVGYALLPLIAGGRMLGPNQHLQLNLLDIEPAMKCLEGIRAELMDCAFPLLDRVVIT---HKPAVAFENVDI
   EcMDH   M-----------------KVAVLGAAGGIGQALALLLKTQLPSGSE----LSLYDIAP---VTPGVAVDLSHIPTAVKIKGF---SGEDATPALEGADV
   ScMDH   MLSRVAKRAFSSTVANPYKVTVLGAGGGIGQPLSLLLKLNHKVTD-----LRLYDLKGA----KGVATDLSHIPTNSVVKGFTPEEPDGLNNALKDTDM
   LpLDH   M----------SSMPNHQKVVLVGD-GAVGSSYAFAMA-QQGIAEE----FVIVDVVKD--RTKGDALDLEDAQAFTAPKKIYS----GEYSDCKDADL

                    #          *                                                                    *
  CvFrsC   VFLISSPLDSLATASKTADAR---MEQHGNTFALHGKALNDVASRDVKILVISNPVMINALMVQRN----APDLNSS-CISALMRLDHNRAHALLAHKA
  BcFrsC   AFLISSPPDSLSEASKTADAR---MENHGATFAVHGRALNGVANRDVKLLVVSNPVMLNALTVQRN----APNLDPSCICGM-MRLDHNRAHALLAHKA
   PfLDH   V-IVTAGFTKAPGKSDKEWNRDDLLPLNNKIMIEIGGHIKKNCPNAF-IIVVTNPVDVMVQLLHQH---SGV--PKNKIIGLGGVLDTSRLKYYISQKL
   TgLDH   V-IITAGLTKVPGKSDKEWSRNDLLPFNAKIIREVAQGVKKYCPLAF-VIVVTNPLDCMVKCFHEA---SGL--PKNMVCGMANVLDSARFRRFIADQL
 WcHicDH   V-ISTLGNIKLQ-QDNPTGDRFAELKFTSSMVQSVGTNLKESGFHGV-LVVISNPVDVITALFQHV---TGF--PAHKVIGTGTLLDTARMQRAVGEAF
 TcAHADH   A-ILCGSFPAKPGTL-----RRDLLQKNAAIFSEHGRLLGELASKDCHVCVVGNPVNTNALVLLNA--SNGKIKPKN--VSALTRLDHNRSLALVAERA
   EcMDH   V-LISAGVARKPGMD-----RSDLFNVNAGIVKNLVQQVAKTCPKAC-IGIITNPVNTTVAIAAEVLKKAGVY-DKNKLFGV-TTLDIIRSNTFVAELK
   ScMDH   V-LIPAGVPRKPGMT-----RDDLFAINASIVRDLAAATAESAPNAA-ILVISNPVNSTVPIVAQVLKNKGVYNPK-KLFGV-TTLDSIRAARFISEVE
   LpLDH   V-VITAGAPQKPGES-----RLDLVNKNLNILSSIVKPVVDSGFDGI-FLVAANPVDILTYATWKF---SGF--PKDRVIGSGTSLDSSRLRVALGKQF

                          *
  CvFrsC   GASLADVRKVIVWGNHSSTQYPDFYHATIGGVRVDALLENDWLHQVSIG----LVRQRGYAVIDAYGGLRAAS-SAAKAAIDHMRDWIFGTRDGDWT-S
  BcFrsC   RVHLSEVRKVIVWGNHSRTQYPDFDHATIGGIPACNLISHDWLRQDSV----DIVRQRGYAVIDAYGGLRAAS-SAAKAAIDHMRDWVFGTREGDWT-S
   PfLDH   NVCPRDVNAHIV-GAHGNKMVLLKRYITVGGIPLQEFINNKLISDAELEAIFDRTVNTALEIVNLH---ASPYVAPAAAIIEMAESYL---KDLKKVLI
   TgLDH   EISPRDIQATVI-GTHGDHMLPLARYVTVSGFPLREFIKKGKMTEAKLAEIVERTKKAGGEIVRLL-GQGSAYYAPALSAITMAQAFL---KDEKRVLP
 WcHicDH   DLDPRSVSGYNL-GEHGNSQFVAWSTVRVMGQPIVTLADAG---DIDLAAIEEEARKGGFTVLN---GKGYTSYGVATSAIRIAKAVM---ADAHAELV
 TcAHADH   NAHVRDVKNCIIWGNHSGTQVPDVNSATVRGVPVREAIKDDAYFD---GEFMTTVQQRGYEIIRWRG--NSSALSAANAAVDQVHDWVLGTPTGTHV-S
   EcMDH   GKQPGEVEVPVI-GGHSG--------VTI--LPLLSQVPGVSFTEQEVADLTKRIQNAGTEVVEAKAGGGSATLSMGQAAARFGLSLVRALQGEQGVVE
   ScMDH   NTDPTQERVNVI-GGHSG--------ITI--IPLISQTNHKLMSDDKRHELIHRIQFGGDEVVKAKNGAGSATLSMAHAGAKFANAVLSGFKGERDVIE
   LpLDH   NVDPRSVDAYIM-GEHGDSEFAAYSTATIGTRPVRDVAKEQGVSDEDLAKLEDGVRNKAYDIINLK---GATFYGIGTALMRISKAIL---RDENAVLP


  CvFrsC   MGVLSDGSY-GIPSGIFFGFPVVADG-GQVNIVQGLQICPERLEKIHHSADEIYRRCQQ-FNLL---  ...328 AA
  BcFrsC   MGVFSDGSY-GVPAGIFFGYPVV-SHQGDLHIVKNLRPNPIVLEKIHLSADEIYQR-SKNFKLI---  ...328 AA
   PfLDH   CSTLLEGQY--GHSDIFGGTPVVLGANGVEQVIEL-QLNSEEKAKFDEAIAETKRM--KALA-----  ...316 AA
   TgLDH   CSVYCQGEY--GLHDMFIGLPAVIGGGGIEQVIEL-ELTHEEQECFRKSVDDVVE-LNKSLAAL--G  ...326 AA
 WcHicDH   VSNRRDDM------GMYLSYPAIIGRDGVLAETTL-DLTTDEQEKLLQSRDYIQQRFDEIVDTL---  ...310 AA
 TcAHADH   MAVYSDGNPYGVPPGLVFSFPVTCSG-GEWQFVENACVTPSVAKHLAATTKELEEERSESLSLA--L  ...332 AA
   EcMDH   CA-YVEGD---GQYARFFSQPLLLGKNGVEERKSIGTLSAFEQNALEGMLDTLKKDIALGEEFV-NK  ...312 AA
   ScMDH   PS-FVDSPLFKSEGIEFFASPVTLGPDGIEKIHPIGELSSEEEEMLQKCKETLKKNIEKGVNFVASK  ...334 AA
   LpLDH   VGAYMDGQY--GLNDIYIGTPAVIGGTGLKQIIES-PLSADELKKMQDSAATLKKVLNDGLAELENK  ...320 AA
```

Figure S8. Full alignment of FrsC (*C. vaccinii* FrsC; QPI18725, „*Candidatus* Burkholderia crenata“ FrsC: KNE75169.1), PfLDH (*Plasmodium falciparum* LDH; Q27743), TgLDH (*Toxoplasma gondii* LDH; Q27797), WcHicDH (*Weissella confusa* HicDH; P14295), TcAHADH (*Trypanosoma cruzi* l-alpha-hydroxyacid dehydrogenase; AAF36775), EcMDH (*Escherichia coli* K-12 MDH, P61889), ScMDH (*Saccharomyces cerevisiae* MDH; NP_012838), LpLDH (*Lactiplantibacillus plantarum* LDH; ACN66626). The length of each enzyme is indicated at the end of the alignment. #: Specificity residue at position 102, *: Conserved residues directly interacting with the substrate.
